# Pressure Sores

**Published:** 2013-01-21

**Authors:** Sachin M. Shridharani, Howard D. Wang, Justin M. Sacks

**Affiliations:** Department of Plastic and Reconstructive Surgery, The Johns Hopkins University School of Medicine, Baltimore, Md

**Figure:**
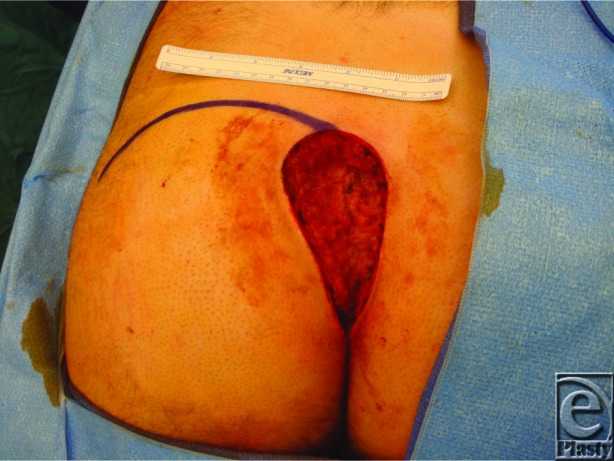


## DESCRIPTION

A 31-year-old man with known history of paraplegia presents to clinic with a long-standing wound as shown earlier. The wound is located in the presacral region with intact periosteum, healthy overlying fascia, and no purulence.

## QUESTIONS

1. **What is the pathogenesis of this wound?**
2. **How would you stage this pressure sore?**
3. **What steps should be taken to diagnose and manage osteomyelitis?**
4. **Which preoperative factors impact the risk of recurrence after surgical coverage?**
5. **What are the surgical options for this patient?**
6. **What measures should be taken to protect the repair and prevent recurrence in the postoperative period?**

## DISCUSSION

This patient has a pressure sore in the sacral region, which is usually the result of prolonged lying in the supine position. Other common areas where pressure sores develop include the ischial region in patients who are sitting and the trochanteric area from patients lying in the lateral decubitus position. Common mechanism in all three is unrelieved pressure over a bony prominence. As the external pressure exceeds the capillary pressure, generally occurring between 12 to 32 mm Hg, tissue ischemia will result over time.^1^

The most commonly used staging system for pressure sores was described by the National Pressure Sore Advisory Panel Consensus Development Conference in 2007. Five stages were described. Stage I is marked by localized nonblanching redness. Stage II can present as a serum-filled blister or a shallow open ulcer, representing partial-thickness loss of dermis. Stage III extends to full-thickness tissue loss with visible subcutaneous fat. In stage IV, bone, tendon, or muscle may be exposed. A fifth stage describes full-thickness tissue loss where slough covers the base of the ulcer, making the true depth of the ulcer difficult to determine and thus unstageable.^2^ In this case, the subcutaneous fat is exposed but the underlying bone and muscles are not, making it a stage III pressure sore.

Osteomyelitis is a common complicating factor in patients with pressure sores.^3^ Prompt diagnosis and adequate management of osteomyelitis is important because it is associated with an increased risk of pressure sore recurrence. The standard for diagnosis of osteomyelitis in patients with pressure ulcers continues to be intraoperative bone biopsy. Preoperative magnetic resonance imaging may be useful as a noninvasive diagnostic tool and to determine the extent of involvement. However, its impact on management and outcomes is unproven.^4^ When a diagnosis of osteomyelitis is made, a combination of bony débridement and antibiotic therapy guided by bone cultures should be employed.^5^

In addition to osteomyelitis, several other preoperative factors contribute to successful coverage of the pressure sore and risk of recurrence after surgical repair. These factors include nutritional status, relief of pressure, control of spasm and contractures, patient mobility, anemia, diabetes mellitus, end-stage renal disease, and cigarette smoking.^1^^,^^6^ Optimization of these factors through nutrition supplementation, controlling medical conditions such as diabetes mellitus, and eliminating tobacco use should be attempted prior to surgical closure.

**Figure 1:**
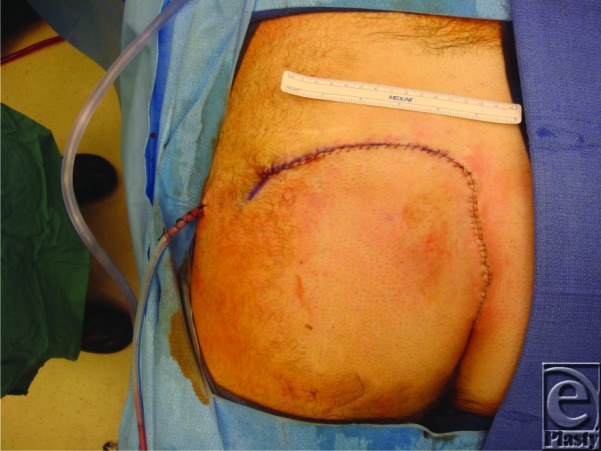
Postoperative image of the patient after coverage with rotational gluteus maximus flap.

Once the challenges of optimizing patient conditions have been achieved, surgical closure can be considered. Closure is most often achieved with pedicled musculocutaneous or fasciocutaneous flaps, while some surgeons advocate the use of free tissue transfer.^7^ For sacral pressure sores, options include rotational gluteus maximus flaps, transverse lumbosacral back flap, and perforator island flaps based on the superior gluteal artery. Ischial defects are often closed with posterior thigh flaps, which can be advanced in a V-Y fashion. Other considerations include posteromedial fasciocutaneous flap, tensor fascia lata flap, and gluteus maximus flap. Flap options for trochanteric ulcers include the tensor fascia lata flap, vastus lateralis flap, and flaps based on the gluteus muscles.^1^ In this case, the rotational gluteus maximus flap was selected and successfully closed the defect (Fig 1).

Despite adequate surgical coverage with vascularized tissue, recurrence rates are extremely high. Average recurrence rates are cited to range from 19% to 90%.^6^ To reduce the rate of recurrence, postoperative measures such as avoiding sitting for at least 3 to 6 weeks after surgery along with the use of periodic repositioning and air fluidized mattress are advocated.^5^^,^^8^ In addition, continued optimization of wound healing by nutrition supplementation, treating infection and managing medical conditions remains essential. Each phase of the management, preoperative, operative, and postoperative, plays an important role in the comprehensive care of patients with pressure sore to achieve the goals of preventing complications, closure of wound, and reducing risk of recurrence.
